# Autonomic dysfunction is associated with disease progression and survival in amyotrophic lateral sclerosis: a prospective longitudinal cohort study

**DOI:** 10.1007/s00415-023-11832-w

**Published:** 2023-06-26

**Authors:** Raffaele Dubbioso, Vincenzo Provitera, Daniela Pacella, Lucio Santoro, Fiore Manganelli, Maria Nolano

**Affiliations:** 1 Department of Neurosciences, Reproductive Sciences and Odontostomatology, University Federico II of Naples, Via Sergio Pansini, 5, 80131 Naples, Italy; 2 Istituti Clinici Scientifici Maugeri IRCCS, Neurological Rehabilitation Unit of Telese Terme Institute, 82037 Telese Terme, Benevento Italy; 3 Department of Public Health, University Federico II of Naples, Naples, Italy

**Keywords:** Autonomic dysfunction, Non-motor symptoms, Survival, Prognosis, ALS, Motor neuron disease

## Abstract

**Background:**

Among non-motor symptoms, autonomic disturbances have been described in amyotrophic lateral sclerosis (ALS) and reported as mild to moderate in up to 75% of patients. However, no study has systematically investigated autonomic symptoms as prognostic factors.

**Objectives:**

The main aim of this longitudinal study was to examine the association of autonomic dysfunction with disease progression and survival in ALS.

**Methods:**

We enrolled newly diagnosed ALS patients and a healthy control group (HC). Time from disease onset to disease milestone (King’s stage 4) and death were calculated to assess disease progression and survival. Autonomic symptoms were assessed by a dedicated questionnaire. Longitudinal evaluation of parasympathetic cardiovascular activity was performed by the heart rate variability (HRV). Multivariable Cox proportional hazards regression models on the risk of the disease milestone and death were used. A mixed-effect linear regression model was used to compare autonomic dysfunction with a HC group as well as its impairment over time.

**Results:**

A total of 102 patients and 41 HC were studied. ALS patients, compared with HC, complained of more autonomic symptoms, especially in bulbar onset patients. Autonomic symptoms occurred in 69 (68%) patients at diagnosis and progressed over time (post-6: *p* = 0.015 and post-12: *p* < 0.001). A higher autonomic symptom burden was an independent marker of faster development of King’s stage 4 (HR 1.05; 95% CI 1.00–1.11; *p* = 0.022); whereas, urinary complaints were independent factors of a shorter survival (HR 3.12; 95% CI 1.22–7.97; *p* = 0.018). Moreover, HRV in ALS patients was lower than in HC (*p* = 0.018) and further decreased over time (*p* = 0.003), implying a parasympathetic hypofunction that progressed over time.

**Conclusion:**

Autonomic symptoms occur in most of the ALS patients at diagnosis and progress over time, implying that autonomic dysfunction represents an intrinsic non-motor feature of the disease. A higher autonomic burden is a poor prognostic factor, associated with a more rapid development of disease milestones and shorter survival.

## Introduction

Although amyotrophic lateral sclerosis (ALS) is traditionally viewed as a neurodegenerative disorder affecting the motor neurons, increasing evidence suggests that ALS should be considered as a multisystem disease with 80% of patients reporting at least one non-motor symptom [1]. Psychiatric, cognitive [2], and behavioral symptoms [3] are most common and associated with shorter survival time and reduced quality of life. Among non-motor symptoms, autonomic dysfunction has been also described in ALS and reported in up to 75% of patients, mild in most patients (85%), and moderate in a minority (15%) [4]. The most experienced symptoms include urinary urgency and frequency in 40% of patients [5, 6] and gastrointestinal dysfunction, most typically constipation, in 50% [7, 8]. Although less disabling to patients, cardiovagal abnormalities, including reduced heart rate variability, are also present in 50% of patients [4, 9], though more serious cardiovascular autonomic failure appears rare and only in more advanced disease stages [10]. Since the autonomic nervous system is responsible for the control of vital functions, its impairment has been consistently associated with shorter survival in several neurological diseases, such as multiple system atrophy [11], Parkinson’s disease [12], dementia with Lewy Body [13] and progressive supranuclear palsy [14]. However, autonomic symptoms have not been systematically assessed as prognostic factors in ALS. Therefore, the main objectives of this study were: first, to investigate in a longitudinal study whether the presence of autonomic symptoms at diagnosis can predict disease progression and survival; and second, to instrumentally assess autonomic dysfunction over time using a simple parasympathetic marker, such as the heart rate variability (HRV) [15].

## Methods

### Study design

We enrolled 102 patients with ALS, newly diagnosed between 1st of January 2018 and 1st of June 2021 at the ALS center of the Federico II University of Naples. All patients included in the study were diagnosed with definite, probable, and probable laboratory-supported ALS according to the revised El Escorial criteria [16]. Exclusion criteria were: (1) comorbidities known to affect the autonomic nervous system (e.g., diabetes or glucose intolerance; dysendocrinopathies; vitamin deficiency; hepatic or renal failure; HIV; dysimmune disorders; heart failure), and (2) genetic forms of ALS (e.g., SOD1, FUS, TARDBP and C9orf72). Forty-one healthy subjects free from any disease or medication potentially affecting the autonomic nervous system served as a control population. This study was approved by the ethics committee of the University Federico II of Naples (N. 100/17) and all participants gave written consent before any testing under the protocol.

### Clinical assessment

At baseline, each patient underwent a complete neurological and neurophysiological examination for diagnosis. Disease severity was assessed using the revised ALS Functional Rating Scale (ALSFRS-R) and respiratory function was assessed through spirometry with the patient sitting upright. Results for forced vital capacity (FVC) were expressed as a percentage of predicted value, from an average of three trials [17]. We then computed the ALSFRS-R median monthly decline (∆ALSFRS-R) using the following formula: (48 − ALSFRS-R score at baseline assessment)/(months from onset to baseline assessment) [18]. Similarly, FVC median monthly decline (∆FVC) as (1.00 − FVC at baseline assessment)/(months from onset to baseline assessment) [19]. We also defined as the main clinical milestone the stage 4 of the modified King’s staging system [20], namely the development of nutritional or respiratory failure. The times from disease onset to the development of disease milestone and death were calculated. Lastly, neuropsychological tests were performed to classify patients according to consensus criteria [21] as having “normal cognition” (i.e., ALS-nc) or “cognitive and/or behavioral impairment” (i.e., ALS with cognitive impairment, ALS-ci; ALS with combined cognitive and behavioral impairment, ALS-cbi; ALS with behavioral impairment, ALS-bi) [22, 23]. A detailed neuropsychological battery has been described elsewhere [24, 25].

### Clinical autonomic assessment

Autonomic symptoms were evaluated at baseline, and at 6 and 12 months of follow-up using the Scales for Outcomes in Parkinson’s Disease-Autonomic Dysfunction (SCOPA-AUT). This questionnaire was developed for patients with Parkinson’s disease [26] and has proved reliable for the screening of autonomic symptoms in other neurodegenerative disorders [27, 28]. The questionnaire is composed of 23 items exploring the occurrence of symptoms during the last month related to the function in six different autonomic domains: gastrointestinal (7 items), urinary (6 items), cardiovascular (3 items), thermoregulatory (4 items), pupillomotor (1 item) and sexual (2 items). For each item, the options regarding the occurrence of the symptom are ‘never’ (score 0), ‘sometimes’ (score 1), ‘regularly’ (score 2) and ‘often’ (score 3). The total score ranges from 0 (no symptoms) to 69 (all symptoms occur often). A domain was considered impaired if at least one item scored ≥ 2. Two items, pertaining to the cardiovascular domain, imply the ability to stand up; for patients unable to assume an upright position, we asked whether the symptoms occurred passing from the supine to the sitting position. Moreover, for gastrointestinal domain, we did not consider the items related to dysphagia (items 1 and 3) and sialorrhea (item 2), to avoid any confounding factor by the underlying motor impairment. At follow-up for patients who were not able to come to the hospital, SCOPA-AUT questionnaire was administered using an online interview by the same-trained interviewer (R.D.) of baseline evaluation. Scale was sent to the patients in advance via mail to prepare them for the interviews. Here is the link for the full English version of the scale https://www.movementdisorders.org/MDS-Files1/PDFs/Rating-Scales/SCOPA-AUT_FINAL.pdf.

### Heart rate variability

The assessment of HRV was conducted in the morning, after a light breakfast and at least 24–48 h after discontinuing therapy affecting autonomic functions (e.g., beta-blockers and anticholinergic drugs).

After resting for at least 10 min, heart rate variability (HRV) was measured under normal breathing for 10 min in the supine position via Dantec Keypoint Workstation (Natus Medical, San Carlos, CA). After acquisition, the signal was visually inspected for ectopic beats. Besides heart rate at rest, time domain analysis of HRV was assessed by the standard deviation of the R–R interval time series (SDRR). SDRR is considered a marker of the parasympathetic activity and a higher value indicates higher variability [15]. SDRR and heart rate recorded at baseline were compared to those obtained in a healthy control (HC) population enrolled among volunteers (Table 1). Longitudinal assessment of SDRR and heart rate was performed only after 6 months, and no further evaluations were performed due to the significant patients drop out at 1 year of follow-up.

**Table 1:** Participants demographic, clinical and autonomic characteristics

	Patients (*N* = 102)	Healthy controls (*N* = 41)	*p* value
	Median (IQR) or *N*. (%)		
Demographic and clinical features			
Age at onset, years	62 (54.5–70)	60 (52–67)	0.11
Gender (female, %)	37 (36.3%)	22 (53.7%)	0.06
Site of onset (bulbar, %)	17 (16.7%)	–	–
Riluzole therapy (yes, %)	93 (91.2%)	–	–
Diagnostic delay (disease onset to diagnosis), months	13 (7–20)	–	–
ALSFRS-R score at diagnosis	37 (34–41)	–	–
ΔALSFRS-R (median points/month)	0.8 (0.4–1.6)	–	–
FVC (%) at diagnosis	88 (72–101.8)	–	–
ΔFVC (median points/month)	0.9 (0.1–2.8)	–	–
Cognition (impaired, %)	40 (39.2%)	–	–
Median follow-up (all participants), months	12 (5–19.8)	–	–
Survival time (disease onset to death), months	21 (16–35)	–	–
Milestone time (disease onset to King’s 4 stage), months	17 (12–28.5)	–	–
Number deceased patients (%)	37 (36.3%)	–	–
Autonomic assessment			
SCOPA-AUT (total score)	10 (4.3–15)	5 (3–6)	** < 0.001**
Gastrointestinal (impaired, %)	43 (42.2%)	8 (19.5%)	**0.01**
Urinary (impaired, %)	44 (43.1%)	7 (17.1%)	**0.006**
Cardiovascular (impaired, %)	9 (8.8%)	1 (2.4%)	0.18
Thermoregulatory (impaired, %)	23 (22.5%)	0 (0%)	**0.001**
Pupillomotor (impaired, %)	11 (10.8%)	0 (0%)	**0.03**
Sexual (impaired, %)	20 (19.6%)	3 (7.3%)	0.07
SDRR (log)	1.4 (1.2–1.6)	1.6 (1.4–1.8)	**0.018**
Heart rate (bpm)	71.8 (60.7–80.2)	64 (60.9–71.3)	**0.032**

Group comparisons were performed by means of non-parametric Mann–Whitney *U* test or Chi-square test as appropriate. In bold significant *p* < 0.05

*ALSFRS-R* revised amyotrophic lateral sclerosis functional rating scale, *FVC* forced vital capacity %, *ΔALSFRS-R* ALSFRS-R median monthly decline, *ΔFVC* FVC median monthly decline, *SCOPA-AUT* scales for outcomes in Parkinson’s disease-autonomic dysfunction, *SDRR (log)* log transformation of standard deviation of the R–R interval time series, *SD* standard deviation, *IQR* interquartile range.

### Statistical analysis

Demographic and clinical characteristics of the participants were reported as percentages for categorical variables and as mean (standard deviation) or median (interquartile range) for parametric and non-parametric continuous variables. Parametric statistical tests were applied to SDRR after log transformation of raw values. Survival time was defined as the time from symptom onset to the time of death. Analogously, milestone time was the time from symptom onset to the development of nutritional and/or respiratory failure (King’s stage 4). Patients who were alive at the time of analysis were censored.

To explore the association of autonomic dysfunction with the risk of developing the disease milestone or the risk of death (survival), simple and multiple Cox proportional hazards regression models were adopted, and *p* values were computed using Wald test. In the Cox models, we considered as clinical variables: age at disease onset, sex (female vs male), disease onset (spinal vs bulbar), disease severity progression rate (ΔALSFRS-R), FVC rate (ΔFVC), cognitive and/or behavioral impairment (yes or no), and autonomic variables: SCOPA-AUT total score, impairment (yes or no) of gastrointestinal, urinary, cardiovascular, thermoregulatory, pupillomotor and sexual domains, SDRR and heart rate.

Survival curves were constructed with the Kaplan–Meier method for the autonomic covariates that were independently associated with disease milestone and survival time according to Cox analysis, and patients were stratified by the median value of the SCOPA-AUT, SDRR and heart rate or the presence/absence of autonomic disturbances in each domain (gastrointestinal, urinary, cardiovascular, thermoregulatory, pupillomotor and sexual). Log-rank test was then applied to compute the differences between the curves.

To compare SDRR and heart rate values between ALS patients and healthy controls, linear regression model was applied considering as covariates age, sex and FVC% (stratifying patients in two groups, FVC ≥ 75% and FVC < 75%) [17]. Finally, a mixed-effect linear regression model was applied for longitudinal analysis of clinical (SCOPA-AUT score) and instrumental (SDRR log value, heart rate) assessment of autonomic functions setting time (baseline vs post-6 vs post-12) and disease onset (bulbar vs spinal onset) as fixed factors.

For all analyses, a two-tailed *p* value < 0.05 was considered statistically significant. Analyses were performed using the statistical software R, version 4.0.3 and SPSS version 22 (SPSS Inc., Chicago, IL).

## Results

A total of 102 consecutive patients with ALS who fulfilled the entry criteria (63.7% were male, and 83.3% had spinal onset) were included, and their demographic and clinical characteristics are summarized in Table 1. The mean disease severity (ALSFRS-R) was 37 (IQR 34–41). The median follow-up time among censored observations was 12 months (IQR 5–25) and 36.2% of patients had died by January 2022, with a median follow-up time among deceased patients of 13 months (IQR 5–17). The overall median estimated survival from onset was 21 months (IQR 16–35). The King’s 4 stage, used as the main disease milestone, was reached by seventy-one patients (69.6%) with a median time from disease onset of 17 months (IQR 12–28.5). Lastly, ALS patients compared to the HC group did not differ for age (*p* = 0.11) and sex (*p* = 0.06).

### Longitudinal assessment of autonomic symptoms

The SCOPA-AUT questionnaire was administered at baseline in 102 patients, at 6 months in 82 patients, in 58 patients at 12 months. At baseline, 68% (69/102) of patients referred autonomic symptoms in at least one domain, a percentage that increased to 84.1% (69/82) and 82.8% (48/58) at 6- and 1-year follow-up, respectively. Compared with controls, ALS patients scored higher in the total SCOPA-AUT (*p* < 0.001), with gastrointestinal (*p* = 0.01), urinary (*p* = 0.006), sudomotor (*p* = 0.001) and pupillomotor (*p* = 0.029) domains being more significantly affected. Conversely, no significant difference was found for cardiovascular (*p* = 0.176) and sexual (*p* = 0.07) domains (Table 1).

Specifically, 43.1% of patients complained of urinary disturbances, with urinary urgency incontinence present in 34.3% of patients and urinary retention in 33.3%. ALS patients complaining of both, urinary urgency incontinence and retention, were 24.5%. Other disabling autonomic symptoms, involving gastrointestinal domain, were mainly constipation or early abdominal fullness (*n* = 42, 41.2%), whereas fecal incontinence was rare (*n* = 1, 1%). Sudomotor and sexual symptoms were reported in about one-fifth of participants, while less frequent were cardiovascular symptoms or oversensitivity to bright light (Table 1). Bulbar onset patients showed a higher overall autonomic score compared to those with spinal onset (*β* = − 8.58, *p* < 0.001), also considering the SCOPA-AUT score without the items related to dysphagia and sialorrhea (*β* = − 5.67, *p* < 0.001).

**Fig. 1:**
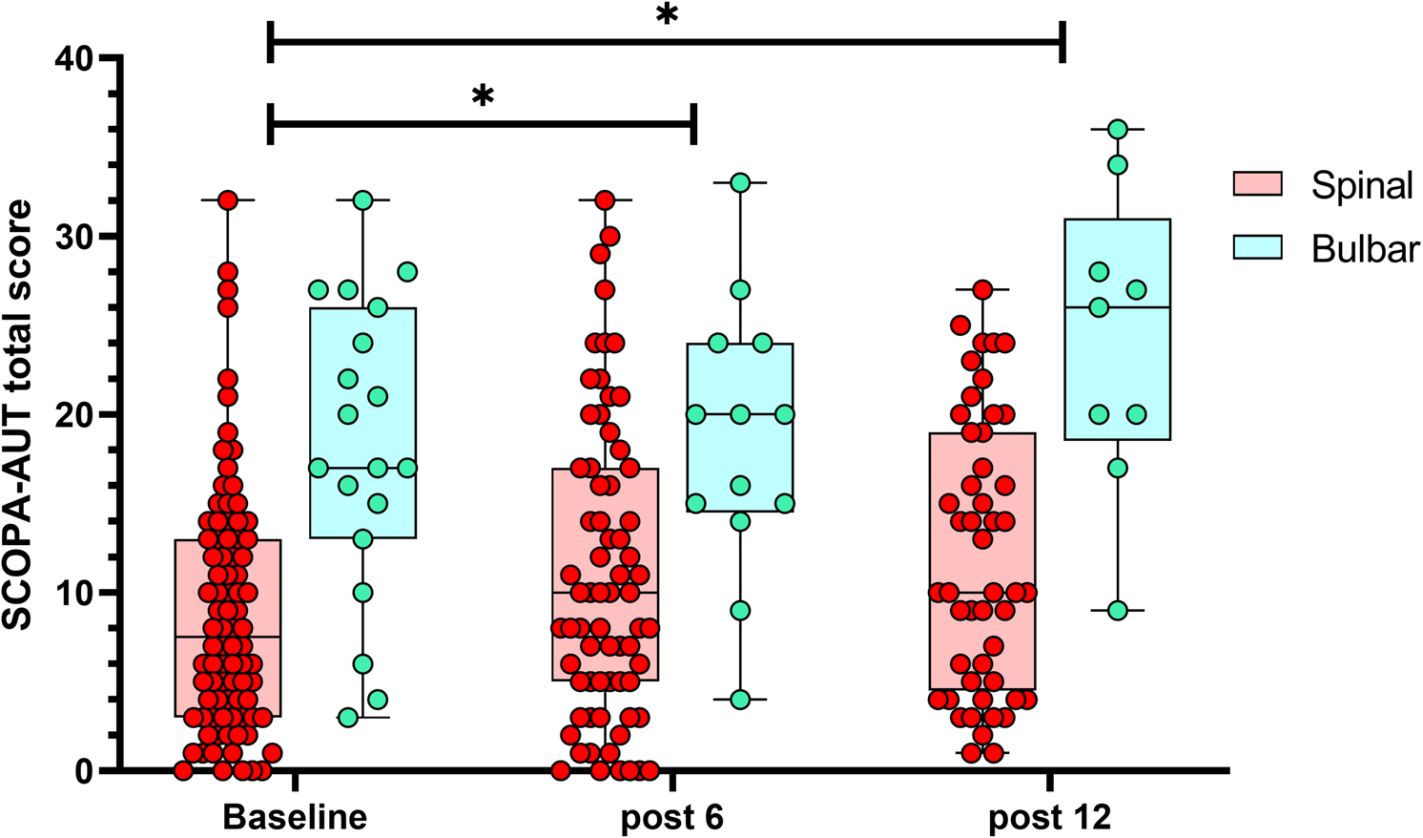
Longitudinal assessment of autonomic symptoms by SCOPA-AUT questionnaire in ALS patients with spinal and bulbar onset. *ALS* amyotrophic lateral sclerosis, *SCOPA-AUT* scales for outcomes in Parkinson’s disease-autonomic dysfunction. *Significant difference of SCOPA-AUT total score at 6 months and 12 months of follow-up compared to the baseline assessment

In the longitudinal study, ALS patients showed a significant increase of SCOPA-AUT total score and the SCOPA-AUT score without dysphagia and sialorrhea at 6 months (*β* = 2.06, *p* = 0.015 and *β* = 1.81, *p* = 0.012) and 12 months of follow-up (*β* = 4.95, *p* < 0.001 and *β* = 4.18, *p* < 0.001) with no difference between bulbar and spinal onset patients in the progression of autonomic impairment over time (all *p* > 0.05), see Fig. 1.

### Association of autonomic dysfunction with disease milestone

**Fig. 2:**
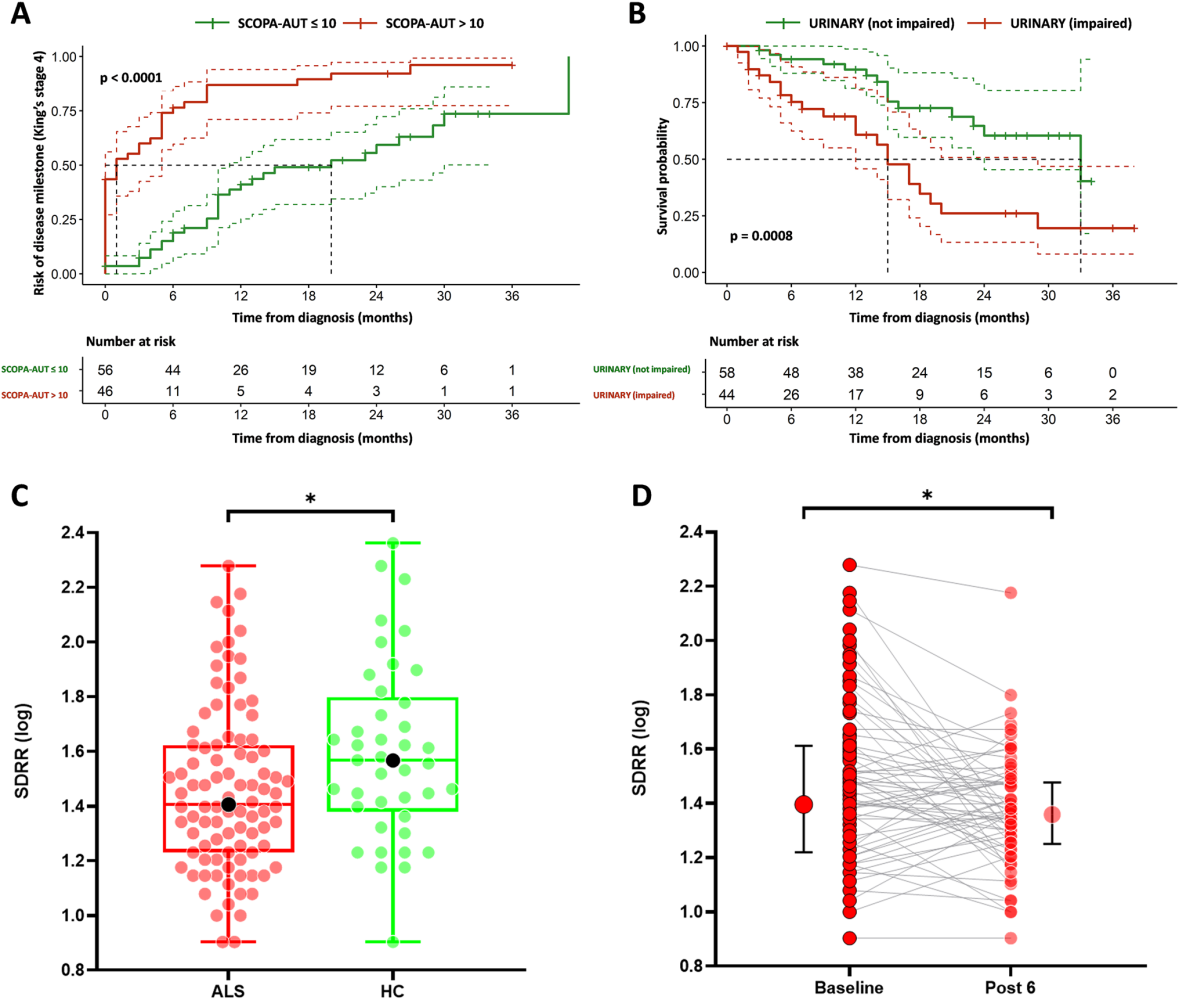
Kaplan–Meier curves of cumulative probability of disease milestone (King’s stage 4) and survival probability among patients with ALS stratified by median value of total SCOPA-AUT score (**A**) and by urinary impairment (**B**). Heart rate variability indexed by SDRR in ALS patients and healthy controls (**C**) and its significant reduction after 6 months (**D**). *ALS* amyotrophic lateral sclerosis, *SCOPA-AUT* scales for outcomes in Parkinson’s disease-autonomic dysfunction, *HC* healthy controls, *SDRR (log)* log transformation of standard deviation of the R–R interval time series

Univariate analysis was used to study the influence of clinical data as well as autonomic factors on the development of the clinical milestone (King’s stage 4). Factors associated with an earlier development of King’s stage 4 included older age at onset, bulbar onset, a faster decline of disease disability and respiratory function, cognitive impairment, a higher overall autonomic dysfunction score, gastrointestinal and urinary impairment. Five of these variables (age at onset, bulbar onset, a faster decline of disease disability and respiratory function, overall autonomic dysfunction score) were maintained in the multivariate analysis as independent determinants of higher risk of reaching the disease milestone (Table 2). Association of autonomic symptoms with the risk of disease milestone was also confirmed by Kaplan–Meier curves of cumulative probability of King’s stage 4, specifically a greater overall autonomic dysfunction (*p* < 0.0001) was associated with a higher probability of reaching the disease milestone (Fig. 2A).

**Table 2:** Multivariable analysis of disease progression predictors

Variable	Crude HR (95% CI)	*p* value	Adjusted HR (95% CI)	*p* value
Clinical covariates				
Age at onset	1.04 (1.02, 1.06)	**0.001**	1.05 (1.02, 1.08)	** < 0.001**
Sex (male)	0.86 (0.67, 1.10)	0.218		
Site of onset (bulbar)	3.38 (1.90, 5.98)	** < 0.001**	2.71 (1.42, 5.18)	**0.003**
ΔALSFRS-R (median points/month)	2.32 (1.88, 2.86)	** < 0.001**	2.11 (1.51, 2.95)	** < 0.001**
ΔFVC (median points/month)	1.38 (1.27, 1.50)	** < 0.001**	1.19 (1.05, 1.34)	**0.004**
Cognition (impaired)	2.56 (1.59, 4.11)	** < 0.001**	1.4 (0.83, 2.36)	0.204
Autonomic covariates				
SCOPA-AUT (total score)	1.07 (1.04, 1.10)	** < 0.001**	1.05 (1.00, 1.11)	**0.022**
Gastrointestinal (impaired)	2.29 (1.43, 3.67)	**0.001**	0.85 (0.45, 1.63)	0.630
Urinary (impaired)	1.15 (0.72, 1.84)	** < 0.001**	1.26 (0.64, 2.49)	0.512
Cardiovascular (impaired)	2.26 (1.01, 5.04)	0.05		
Thermoregulatory (impaired)	1.7 (0.95, 3.03)	0.07		
Pupillomotor (impaired)	0.59 (0.28, 1.26)	0.17		
Sexual (impaired)	1.54 (0.89, 2.67)	0.12		
SDRR (log)	0.83 (0.34, 2.03)	0.68		
Heart rate (bpm)	1.00 (0.98, 1.03)	0.44		

In bold significant *p* < 0.05

*ALSFRS-R* revised amyotrophic lateral sclerosis functional rating scale, *FVC* forced vital capacity %, *ΔALSFRS-R* ALSFRS-R median monthly decline, *ΔFVC* FVC median monthly decline, *SCOPA-AUT* scales for outcomes in Parkinson’s disease-autonomic dysfunction, *SDRR (log)* log transformation of standard deviation of the R–R interval time series

### Association of autonomic dysfunction with survival

The association of clinical and autonomic data with survival is summarized in Table 3. Specifically, we found that clinical factors such as older age at onset, bulbar onset, a faster decline of disease disability and respiratory function, cognitive impairment at diagnosis were associated with a shorter survival. Interestingly, autonomic factors such as a higher score at SCOPA-AUT at baseline, gastrointestinal and urinary disturbances were also associated with a poor outcome. Only three variables (older age, faster decline of disease disability and urinary disturbances) survived in the multivariate analysis as independent determinants of shorter survival. Kaplan–Meier curves of survival probability confirmed that urinary impairment at diagnosis (*p* = 0.0008) was a prognostic factor of poor outcome (Fig. 2B).

**Table 3:** Multivariable analysis of survival predictors

Variable	Crude HR (95% CI)	*p* value	Adjusted HR (95% CI)	*p* value
Clinical covariates				
Age at onset	1.05 (1.02, 1.09)	**0.003**	1.08 (1.04, 1.12)	** < 0.001**
Sex (male)	1.13 (0.47, 1.77)	0.775		
Site of onset (bulbar)	2.16 (1.02, 4.62)	**0.046**	2.0 (0.72, 5.6)	0.19
ΔALSFRS-R (median points/month)	2.42 (1.79, 3.25)	** < 0.001**	2.7 (1.6, 4.57)	** < 0.001**
ΔFVC (median points/month)	1.37 (1.23, 1.52)	** < 0.001**	1.13 (0.99, 1.3)	0.09
Cognition (impaired)	2.33 (1.21, 4.52)	**0.012**	0.69 (0.3, 1.6)	0.37
Autonomic covariates				
SCOPA-AUT (total score)	1.05 (1.01, 1.09)	**0.018**	0.96 (0.89, 1.03)	0.228
Gastrointestinal (impaired)	2.04 (1.06, 3.91)	**0.032**	1.02 (0.43, 2.46)	0.959
Urinary (impaired)	2.60 (1.34, 5.02)	**0.005**	3.12 (1.22, 7.97)	**0.018**
Cardiovascular (impaired)	2.14 (0.29, 15.7)	0.453		
Thermoregulatory (impaired)	1.76 (0.86, 3.59)	0.12		
Pupillomotor (impaired)	2.18 (0.9, 5.27)	0.085		
Sexual (impaired)	1.56 (0.77, 3.16)	0.221		
SDRR (log)	0.98 (0.28, 3.46)	0.971		
Heart rate (bpm)	1.03 (0.99, 1.062)	0.108		

In bold significant *p* < 0.05

*ALSFRS-R* revised amyotrophic lateral sclerosis functional rating scale, *FVC* forced vital capacity %, *ΔALSFRS-R* ALSFRS-R median monthly decline, *ΔFVC* FVC median monthly decline, *SCOPA-AUT* scales for outcomes in Parkinson’s disease-autonomic dysfunction, *SDRR (log)* log transformation of standard deviation of the R–R interval time series

### Instrumental assessment of cardiac parasympathetic activity

ALS patients compared to HC showed a significantly higher heart rate at rest (*β* = − 5.0, *p* = 0.032), mostly in patients with respiratory impairment (*β* = 7.5, *p* = 0.009), in contrast the SDRR values were significantly lower in patients (*β* = 0.14, *p* = 0.018), with no effect of respiratory dysfunction (*β* = − 0.11, *p* = 0.121), Table 1 and Fig. 2C.

Conversely to autonomic scale, SDRR was assessed in 92 patients at baseline and in 58 at 6 months of follow-up; further instrumental autonomic evaluation was prevented by accrued physical disability, death, or the desire of the patient to discontinue participation. The mixed linear regression model showed a significant reduction of SDRR at 6-month follow-up (*β* = − 0.100, *p* = 0.003), Fig. 2D, with no difference between spinal and bulbar onset patients (*β* = 0.055, *p* = 0.423). No significant change for heart rate was observed over time (71.8, IQR 60.7–80.2 vs 69.8, IQR 63.2–80.3; *β* = 1.003, *p* = 0.459) and between spinal and bulbar onset patients (*β* = 4.271, *p* = 0.24).

## Discussion

This is the first prospective and longitudinal study evaluating the autonomic burden at diagnosis and over time in a well-selected and characterized cohort of newly diagnosed ALS patients.

The main finding was that autonomic symptoms were present in most ALS patients at diagnosis, with a higher load in bulbar phenotype. The severity of autonomic symptoms progressed over time, regardless of the clinical phenotype at onset, and was an independent predictor of a more rapid progression of disease disability. Among the autonomic domains, urinary complaints were associated with shorter survival. Parasympathetic dysfunction, indexed by a lower HRV, was present at diagnosis independent of respiratory impairment and of disease onset phenotype and progressed over time.

Autonomic impairment in ALS, analogously to motor neuron dysfunction, is likely resulting from a combination of degeneration within central and peripheral structures. Specifically, central degeneration is supported by pathological studies showing damage in the thoracic [29, 30] and sacral [31] intermediolateral nucleus of the spinal cord, which convey sympathetic and parasympathetic preganglionic neurons, respectively. Peripheral autonomic damage is also plausible since a small fiber pathology, involving somatic and autonomic cutaneous nerves has been described in ALS in the last decade [8, 32-34]. Interestingly, autonomic denervation was associated with vascular remodeling and disease progression rate [8].

Previous literature on autonomic symptoms in ALS was mostly based on retrospective studies, while longitudinal studies in newly diagnosis patients are lacking.

In our cohort, about two thirds of patients at diagnosis complained of autonomic disturbances that were more severe in bulbar onset patients. In this subgroup of patients, the higher autonomic load may also be linked to the spatial contiguity of crucial autonomic nuclei involved in gastrointestinal, urinary, and cardiorespiratory function in the brainstem [35]. Post-mortem studies in SOD 1 patients with autonomic failure have reported neuronal degeneration of autonomic structures in the brainstem [36], and therefore similar studies should be conducted in sporadic ALS patients with bulbar onset.

Regardless of the disease onset phenotype, the most experienced symptoms were gastrointestinal disturbances, mainly constipation or early abdominal fullness, as well as urinary symptoms such as urgency/incontinence or retention observed in more than 40% of patients. Significant gastrointestinal symptoms, such as constipation, abdominal pain, nausea and feeling of fullness, have been previously reported with constipation being the most frequent symptom [4, 7, 8, 37]. Interestingly, two studies of patients with ALS identified a significant proportion with delayed gastric emptying [38] and prolonged colonic transit times [39].

Analogously, urinary symptoms have been reported in the ALS population [4, 8], with urinary urgency described in percentages ranging from 20% [4] to 76% [7] of the cases and retention in 41% of patients [5].

Notably, urodynamic studies typically revealed a neurogenic bladder resulting from detrusor sphincter dyssynergia, and increased post-void residual bladder volumes in patients with greater lower limb spasticity, implicating suprasacral pathology [5, 6]. However, the association between clinical disability or disease phenotype and urinary symptoms was not confirmed in a subsequent study [40], suggesting that increased spasticity and or reduced mobility may not be a major explanation for urinary impairment. Furthermore, previous studies failed to demonstrate an impairment of motor neurons that supply the pelvic floor muscles [41].

The association of autonomic dysfunction with disease progression in ALS patients is in line with previous studies in other neurodegenerative diseases [11–14]. We also observed that ALS patients who complained of urinary disturbances at diagnosis had a poor survival suggesting that autonomic impairment has a role in blunting adaptive responses reducing life expectancy of ALS patients.

This result agrees with the findings of a recent cross-sectional study that showed that early and severe neurogenic bladder, demonstrated by urodynamic study in motor neuron disease patients was associated with a reduced survival [40], regardless of their phenotype, suggesting a more rapid spread of the disease to extra-motor networks in this patients’ subgroup.

Autonomic dysfunction in our patients was also confirmed by the significant reduction of the SDRR, an index of decreased cardiovagal tone, together with an increase of heart rate indicating sympathetic overactivity. These results are in keeping with previous studies [4, 9, 42–44] that suggest an imbalance between enhanced sympathetic and reduced parasympathetic drives [44] in the autonomic cardiovascular state of ALS patients. Nevertheless, our results showed that the cardiac sympathetic hyperactivity was driven by pulmonary capacity, whereas the impairment of the parasympathetic tone was independent of respiratory dysfunction as well as of the disease onset phenotype. Moreover, the longitudinal study of autonomic cardiac assessment revealed that only parasympathetic hypofunction progressed over time, whereas sympathetic overactivity tended to remain stable and therefore it appeared unrelated to the reduction of parasympathetic drive. Importantly, the impairment of parasympathetic tone did not correlate with autonomic symptoms, demonstrating that it did not reflect the overall autonomic burden. So, it was not surprising that there was no correlation between the HRV and the aggressiveness of the disease. Furthermore, cardiovascular symptoms were not among the most frequently reported by our patients.

In this study, we could not assess any potential effect of riluzole on the cardiac autonomic impairment, since most of patients were on treatment; however, there is no evidence that riluzole affects the heart rate [45].

In conclusion, our study shows that the presence of autonomic symptoms at diagnosis is an independent factor of disease progression and highlights the concept that early involvement of non-motor networks can significantly predict the clinical picture and prognosis, as it is associated with more rapid rates of motor functional decline and shorter survival. This may imply that early autonomic impairment is the expression of a more aggressive multisystemic degenerative process; however, we cannot exclude that autonomic dysfunction by itself may further affect the disease course.

Lastly, our findings have important clinical implications: clinicians should identify autonomic symptoms as part of the routine clinical evaluation and adopt therapeutic approaches to counteract the effect of autonomic dysfunction on disease, thus improving survival and quality of life of patients with ALS.

Further studies of ALS populations including larger numbers of patients with bulbar phenotype are needed. Moreover, the functional assessment of each autonomic domain, especially the most involved ones such as urinary and gastrointestinal, may help to tailor treatment strategies and objectively monitor autonomic dysfunction.

## Data Availability

The dataset supporting the conclusions of the manuscript will be made available by the authors, to any qualified researcher, without breaching participant confidentiality.
